# Chemical Evaluation, *In Vitro* and *In Vivo* Anticancer Activity of *Lavandula angustifolia* Grown in Jordan

**DOI:** 10.3390/molecules27185910

**Published:** 2022-09-12

**Authors:** Nour H. Aboalhaija, Heba Syaj, Fatma Afifi, Suhair Sunoqrot, Eveen Al-Shalabi, Wamidh Talib

**Affiliations:** 1 Department of Pharmaceutical Sciences, Faculty of Pharmacy, Al-Zaytoonah University of Jordan, Amman 11733, Jordan; 2 Department of Pharmaceutical Chemistry and Pharmacognosy, Faculty of Pharmacy, Applied Science Private University, Al-Arab Str. 21, Amman 11931, Jordan; 3 Department of Clinical Pharmacy and Therapeutics, Faculty of Pharmacy, Applied Science Private University, Al-Arab Str. 21, Amman 11931, Jordan

**Keywords:** *Lavandula angustifolia* Mill., Lamiaceae, herbal medicine, antioxidant, breast cancer

## Abstract

*Lavandula angustifolia* is the most widely cultivated *Lavandula* species for medicinal use. In this study, chemical and biological evaluation of *L. angustifolia* aqueous, methanol (MeOH), ethanol (EtOH), ethyl acetate (EtOAc), and chloroform (CHCl_3_) extracts were conducted. Phytochemically, the extracts’ total phenol and flavonoid contents and their antioxidant potential were evaluated. Ethanol extract was analyzed by LC-MS. All extracts were screened *in vitro* for their antitumor potential using human breast cancer cell lines MCF-7 and MDA-MB-23. For the first time, the antiproliferative potential of the EtOH extract was tested in vivo using mice with induced breast cancer. Ethanol extract exhibited the best cytotoxicity and safety profile of the tested extracts, with IC_50_ values of 104.1 µg/mL on MCF-7 and 214.5 µg/mL on MDA-MB-231 cell lines, respectively. *In vivo*, this extract revealed a reduction in tumor size by 43.29% in the treated group, compared to an increase in the tumor growth by 58.9% in the control group. Moreover, undetected tumor was found in 12.5% of the sample size. In conclusion, this study provides novel insight and evidence on the antiproliferative efficacy of *L. angustifolia* ethanol extract against breast cancer with potent anti-oxidant potential.

## 1. Introduction

With more than 200 different types, cancer is the most prevalent disease, affecting more than 60 organs in the human body and with a high mortality rate [1]. An estimated 19.3 million new cases of cancer were diagnosed in 2020 worldwide, causing around 10.0 million deaths. Female breast cancer is the most often diagnosed malignancy, followed by lung, colorectal, prostate, and stomach cancers. In 2040, the worldwide cancer burden is predicted to reach 28.4 million cases [2]. Medicinal plants are currently considered a valuable source for new anticancer agents due to their potent antioxidant activities, antimutagenic properties, their low cost, low side effects, and their easy accessibilities [3].

*Lavandula angustifolia* Mill. (Lamiaceae), formerly known also as *L. vera*, is the most common species of the genus *Lavandula,* growing in Jordan. The name “angustifolia” means “narrow leaf” in Latin. Earlier it was referred to as *L. officinalis*, which denoted its therapeutic characteristics. Its popular names include true lavender, common lavender, English lavender, and narrow-leaved lavender, as well as garden lavender [4]. It is a frost-hardy species with a variety of attractive cultivars, bloom colors, and habits [5]. A broad spectrum of biological activities has been reported for the *L*. *angustifolia* essential oil and for the different extracts of lavender to justify the widespread use of this species in medicine and cosmetics [6,7,8]. Among other potential activities, such as antimicrobial, anti-inflammatory, sedative, antidepressive, and aphrodisiac activities, the antiproliferative effect of the essential oil and extracts has also been screened using different cell lines [9,10,11]. Using three breast cancer cell lines (MCF7, T47D, and ZR-75-1), the antiproliferative activity of the *L. angustifolia* ethanol extract and essential oil was evaluated by Afifi et al. (2016) [12]. The ethanol extract exhibited potent antiproliferative activity with minimum toxicity towards the normal fibroblast [12]. The essential oil and three different extracts (EtOH, n-hexane, and water), prepared from *L. angustifolia* and grown in Iran, were tested for their antiproliferative activity using breast (MCF7) and human cervix carcinoma (HeLa) cell lines. This study revealed that EtOH and n-hexane extracts and the essential oil of *L. angustifolia* inhibited the cell proliferation of HeLa and MCF-7 cells *in vitro* via inducing apoptosis [13]. On the other hand, the antiproliferative activity of the essential oil against lung (A549 and H1299) and glioma (C6) cancer cells through induction of both apoptosis and necrosis has been reported [14]. A weak antiproliferative activity was reported by Alexa et al. (2018) for the aromatic water of *L. angustifolia* against breast carcinoma (MDA-MB-231) and human melanoma cell line (A375) [15]. Recently, Fahmy et al. (2022) evaluated the anticancer activity of lavender essential oil against six human cancer cell lines, namely, hepatocellular- (HepG2), prostate- (PC3), lung- (A549), skin- (A431), colon- (HCT116) and breast- (MCF7) cancer [8]. Researchers observed a highly cytotoxic effect on HepG2 and A549 cell lines. Earlier, Kozics et al. (2017) demonstrated in *in vitro* and *ex vivo* experiments using HepG2 cell lines, the DNA protective efficacy of the lavender essential oil [11]. Although these *in vitro* studies have highlighted the anticancer potential of *L*. *angustifolia* essential oils and extracts against the tested cancer cell lines, to the best of our knowledge, no reported *in vivo* studies confirmed this potential of lavender extracts on breast cancer [12,13,14,15,16,17]. Hence, this work aimed to evaluate the *in vitro* and *in vivo* antiproliferative activities of different extracts of this species, grown in Jordan with emphasis on their antioxidant potency.

## 2. Results and Discussion

### 2.1. Plant Samples Preparation and Percentage Yields

**Figure 1:**
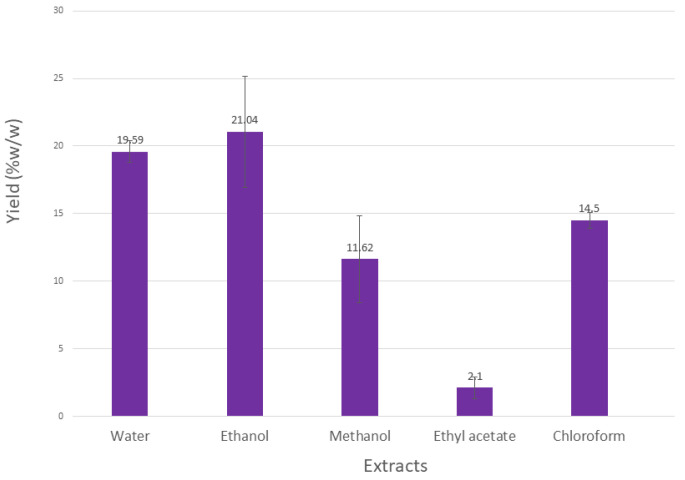
Percentage yields obtained by extraction of 100 g of *L. angustifolia*.

Upon extracting 100 g of *L. angustifolia* aerial parts, the results revealed variations in quantities and % yields. The highest percentage yield was obtained for the EtOH extract, followed by water and MeOH extracts, while EtOAc gave the lowest percentage yield, as shown in Figure 1.

### 2.2. Determination of Total Phenol and Total Flavonoid Contents

The total phenol contents of the aqueous, EtOH, MeOH, EtOAc, and CHCl_3_ extracts for *L. angustifolia* were determined using the Folin-Ciocalteu’s method and expressed in terms of GAE. The standard curve equation is Y = 0.0052X + 0.0024, R^2^ = 0.9996.

The total phenol contents of all tested extracts of *L. angustifolia* are shown in Table 1. Methanol extract revealed the highest total phenol content, while CHCl_3_ extract exhibited the lowest, indicating the polarity of the occurring phenolic compounds.

The total flavonoid contents were determined using the spectrophotometric method with AlCl_3_. Rutin equivalents (the standard curve equation: Y = 0.0152X − 0.0112, R^2^ = 0.9982) were used to express the flavonoid content (Table 1). As expected, the polar extracts showed the highest flavonoid contents, which could be linked with the high phenolic compounds’ concentration.

Similar findings, with slight differences for the total phenol and flavonoid contents, were found in the literature using *L. angustifolia* extracts. While several studies were carried out with water and MeOH extracts of *L. angustifolia*, the current study is the first study evaluating the phenol and flavonoid contents of the EtOH, EtOAc, and CHCl_3_ extracts [18,19,20,21]. The slight differences detected in the phenol and flavonoids contents obtained in the current study compared to the previous ones could be explained by the differences in the collection sites of the plant materials (biogeographic effect), time of collection, and other environmental factors such as soil type and composition, rainfall, seasonal temperature differences, and humidity. Also, the age of the plants and parts used for the extraction are essential parameters, which can affect the actual composition and activity of the extracts, in addition to the experimental conditions [21,22,23,24].

### 2.3. Antioxidant Activity

**Figure 2:**
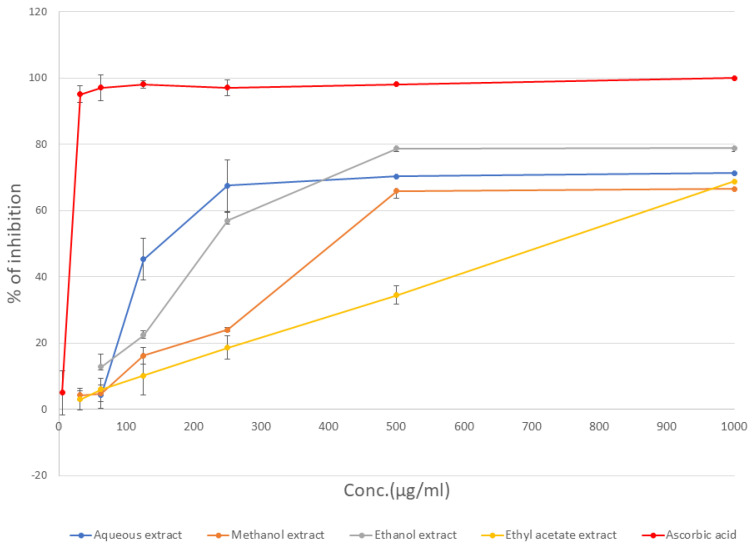
Antioxidant activity of *L. angustifolia* as percentage of DPPH radicals’ inhibition.

The antioxidant activities of different *L. angustifolia* extracts were calculated and expressed in terms of the percentage of DPPH radicals’ inhibition (Figure 2). The calculated IC_50_ values (μg/mL), expressed as average ± SD for triplicate experiments, are shown in Table 2.

As expected, the extracts with high phenol and flavonoid contents (aqueous, MeOH, and EtOH extracts) exhibited potent antioxidant activities. Similar findings were reported by other researchers for *L. angustifolia* aqueous extracts, confirming the potent antioxidant activity of this plant [18,25,26].

### 2.4. Liquid Chromatography-Mass Spectrometry (LC-MS)

Qualitative analysis of the EtOH extract using LC-MS revealed the presence of three compounds, namely, luteolin-7-O-glucoside and rosmarinic and myristic acids after comparing the extract contents with 59 standard references of different classes of secondary metabolites. The LC-MS specifications of the identified compounds are given in Table 3.

A survey of the literature indicated that only one single study was conducted on *L. angustifolia* oil, identifying hydroxycinnamic acids constituents. Hence, this is the first LC-MS analysis of *L. angustifolia* EtOH extract [15].

### 2.5. Antiproliferative Activity

#### 2.5.1. *In Vitro*

**Figure 3:**
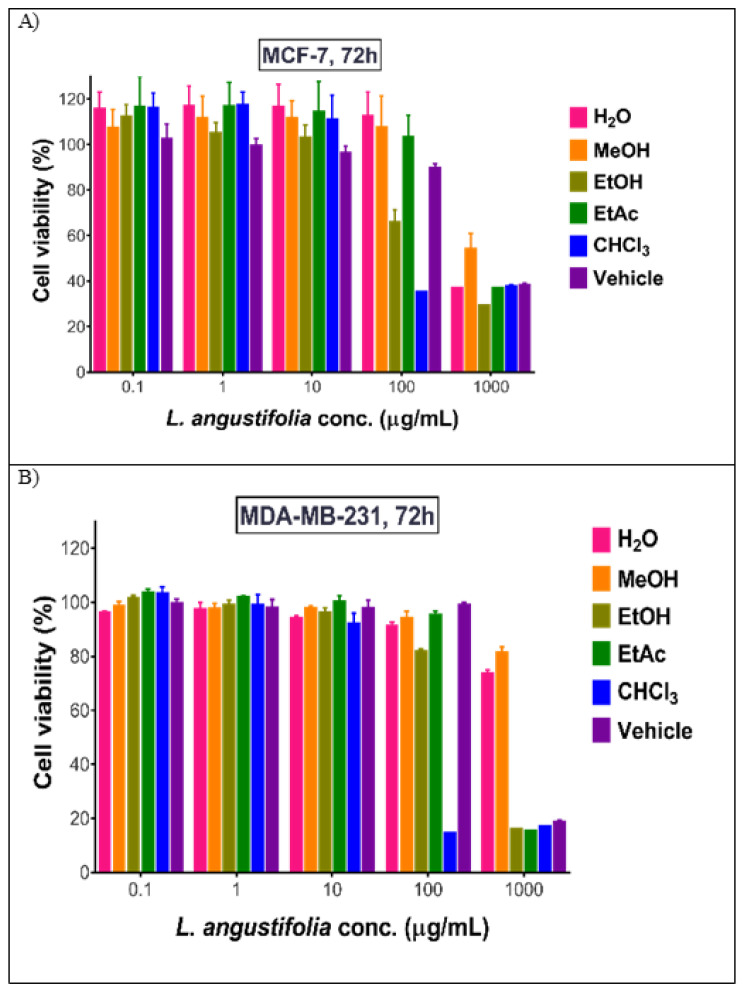
Antiproliferative effect of *L. angustifolia* extracts on (**A**) MCF-7 cells and (**B**) MDA-MB cells. Data are described as mean± SD, *n* = 3.

All prepared *L. angustifolia* extracts were tested for their antiproliferative activities against two breast cancer cell lines, MCF-7 and MDA-MB231, in concentrations ranging between 0.01–1000 µg/mL. As shown in Table 4 and Figure 3, *L. angustifolia* CHCl_3_ extract was found to have the highest cytotoxic activity towards both cell lines followed by EtOH, EtOAc, and MeOH extracts. The latter extract showed activity on MDA-MB231 but not on MCF-7. Aqueous extract leaked the cytotoxic activity compared to the other extracts. All studied extracts tested on the normal fibroblast cells showed safety and selectivity. Except for the CHCl_3_ extract, all studied extracts were found to be safe (Table 4).

Earlier, researchers from Jordan and Iran observed antiproliferative activity of the EtOH extract of *L. angustifolia* against MCF-7 cell lines with similar IC_50_ values [12,13]. Contrary, Hanachi et al. (2021) reported aqueous, MeOH, and EtOH extracts IC_50_ values of more than 1000 µg/mL for HeLa, OVCAR-3, and MCF-7 cancer cell lines [17]. Alexa et al. (2018) observed an inhibition of 14.52% for MDA-MB-231 cell lines by using the aqueous extract of *L. angustifolia* [15]. In the present study, the CHCl_3_ and EtOAc extracts were for the first time evaluated for their antiproliferative activities.

#### 2.5.2. *In Vivo*

Taking into consideration the cytotoxicity and the safety profiles of the tested extracts, EtOH extract was chosen for the *in vivo* experiments against breast cancer because at the concentration of 5 g/kg and by increasing it by 1.5 times, the EtOH extract did not show any mortalities or disabilities. Consequently, 16 female Balb/C mice were injected intraperitoneal (IP) with EMT6/P tumor, and divided into two groups, G1 the control group (not treated) and G2 (treated group). They were then given orally 500 mg/kg/day *L. angustifolia* EtOH extract. Tumor sizes were measured at the beginning of the experiment, at the middle, and at the end, and compared to the control group findings. At the end of the experiment, the tumor was isolated and weighted and compared to the control group.

**Figure 4:**
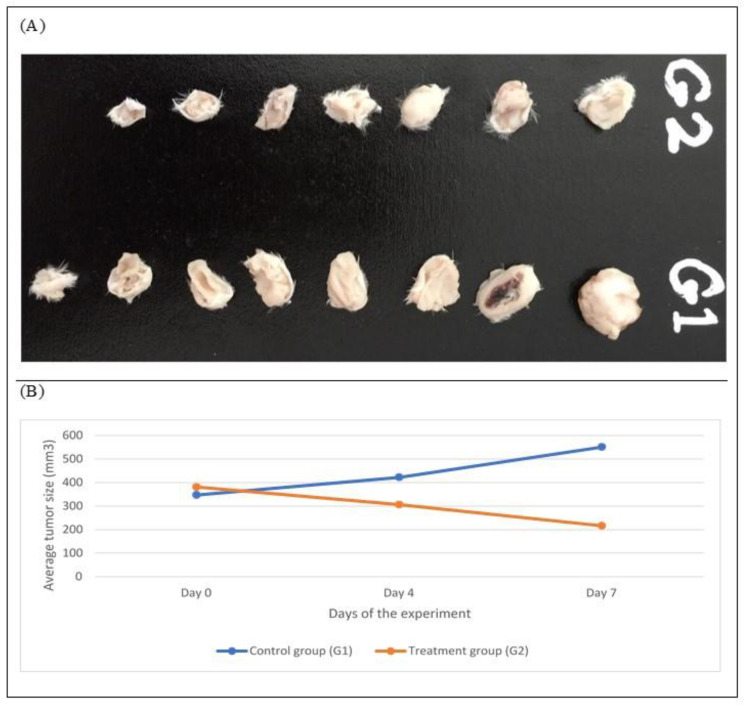
(**A**) Comparison of tumor sizes after dissection at day 7 in all groups, *n* = 8. G1 = Control, G2 = Treated. (**B**) Treatment progression represented as mice’s average tumor size (mm3) over time.

Comparison of tumor size and weight after 7 days of the experiment clearly indicated a significant reduction in tumor size (*p* < 0.01; Table 5 and Figure 4). In the treatment group (G2), the tumor size was decreased by 43.29%, while in the control group (G1) the tumor growth was increased by 58.9%. An undetected tumor was found in one mouse of the treated group, which represents 12.5% of the sample size.

Such regression in tumor size could result from the presence of effective anticancer agents. In the literature, only a single *in vivo* study for *L. angustifolia* is available, in which the essential oil was evaluated for its antiproliferative activity against prostate cancer. The study concluded that the essential oil significantly suppresses the tumor growth. However, to the best of our knowledge, the present study is the first *in vivo* study that was conducted to evaluate the antiproliferative activity of *L. angustifolia* EtOH extract on breast cancer cell lines.

### 2.6. Evaluation of Liver and Kidney Functions

Liver enzyme (AST and ALT) levels were used as indicators of the liver toxicity. Serum levels of liver enzymes were measured for the treated group with *L. angustifolia* (500 mg/kg) and for the control groups. Serum levels were also measured for normal mice with no tumors (as a reference for normal liver function). The treated group showed insignificant (*p* > 0.5) differences in serum ALT and AST levels compared to normal untreated mice as shown in Table 6.

Creatinine levels were measured to assess the possibility of kidney toxicity development after treatment with the EtOH extract of *L. angustifolia*. No significant differences were observed in the creatinine concentration of the treated group compared to the control group or to the normal mice values, which served as a reference (*p* > 0.5) (Table 6). Accordingly, EtOH extract of *L. angustifolia* at the used concentration of 500 mg/kg/day can be considered safe.

## 3. Materials and Methods

### 3.1. Plant Material

The aerial parts (including flowers, leaves, and stems) of *L. angustifolia* were collected in August 2021 from the campus of The University of Jordan (Amman, Jordan). The plant material was authenticated by Prof. Fatma Afifi using descriptive references and by comparison with the herbarium specimen at The University of Jordan (LAM-LA1/FMJ) [27].

### 3.2. Extracts Preparation

To prepare the aqueous and ethanol extracts, each 100 g of dried plants were extracted with 70% EtOH or distilled water, respectively (1:10 *w/v*) by gentle heating until boiling. The extracts were kept overnight at room temperature (RT) and then filtrated. Solvents were evaporated using a rotary evaporator at 40 °C until dry extracts were obtained. To prepare CHCl_3_, EtOAc, and MeOH extracts, 100 g of dried and finely crushed aerial parts of *L. angustifolia* were extracted using a Soxhlet apparatus using each 500 mL CHCl_3_, MeOH, and EtOAc, separately. The extracted solvents were dried using a rotary evaporator and samples were kept refrigerated at 4 °C.

### 3.3. Total Phenol and Total Flavonoid Content

Total phenol content of *L. angustifolia* extracts was determined according to the Folin-Ciocalteu procedure [28]. Briefly, 1 mL of 1 mg/mL of the extract was diluted with 10 mL distilled water in a 25 mL volumetric flask, followed by the addition of 1 mL of Folin-Ciocalteu reagent. After 5 min, 4 mL of 20% sodium carbonate was added and the volume was adjusted to 25 mL with distilled water, incubated in a dark place at RT for 1 h, and then the absorbance was measured at 765 nm. Gallic acid in methanol was used as a calibration curve standard, using five serial dilutions (100, 50, 25, 12.5, and 6.125 μg/mL). Each reading was taken in triplicates and MeOH was used as a blank. The total phenol content was expressed as a gallic acid equivalent (GAE).

Aluminum chloride was used to determine the total flavonoids content [29]. Briefly, 2 mL of 1 mg/mL of the extract was mixed with 0.1 mL AlCl_3_ (10%), 0.1 mL sodium acetate (1M), and 2.8 mL distilled water. The absorbance was measured at 415 nm after incubation for 30 min at RT. Rutin was used as the calibration curve standard using five different concentrations (100, 50, 25, 12.5, and 6.125 μg/mL) in MeOH. The estimation of total flavonoids in the extracts was carried out in triplicates, and the results were then averaged.

### 3.4. Anti-Oxidant Activity

Antioxidant activity of each extract was determined based on the radical scavenging effect of the stable free radical DPPH [30]. Using 96-well plates, in each well, 250 μL of the plant extract in 10 serial concentrations (1–500 μg/mL) was mixed with 40 μL of 0.1 mM MeOH solution of DPPH. Methanol was used as a control. The changes in the absorbance of the samples were measured at 517 nm after 30 min incubation at RT. Trolox was used as standard in 1–500 μg/mL concentrations. The percentage of inhibition of DPPH activity (*I*%) was calculated using the following equation.

*I*% = (*OD_control_* − *OD_sample_*)/*OD_control_* × 100% (1)

where *OD_control_* and *OD_sample_* are the optical densities of the control and the sample, respectively.

### 3.5. Liquid Chromatography-Mass Spectrometry (LC-MS)

A Bruker Daltonik (Bremen, Germany) Impact II ESI-Q-TOF System equipped with Bruker Daltonik (Bremen, Germany) was used for screening compounds of interest, using direct injection. Standards were used for the identification of *m/z* with high-resolution Bruker TOF MS. The instrument was operated using the Ion Source Apollo II ion Funnel electrospray source. The capillary voltage was 2500 V, the nebulizer gas was 2.0 bar, the dry gas (nitrogen) flow was 8 L/min, and the dry temperature was 200 °C. The mass accuracy was ˂1 ppm, the mass resolution was 50,000 FSR (Full Sensitivity Resolution), and the TOF repetition rate was up to 20 kHz. Elute UHPLC coupled to a Bruker impact II QTOFMS chromatographic separation was conducted using a Bruker solo 2.0_C-18 UHPLC column (100 mm × 2.1 mm × 2.0 μm) at a flow rate of 0.51 mL/min and a column temperature of 40 °C as described earlier [29].

Stock solutions were prepared by dissolving the appropriate amount of substance in analytical grade Dimethyl sulfoxide (DMSO), then diluted with Acetonitrile (LC/MS grade), and used for the identification of exact MS. The identification of the compounds in the analyzed extract was based on the comparison of their mass spectrum with the built-in library that includes more than 60 natural compounds.

### 3.6. In Vitro Evaluation of the Antiproliferative Activity

#### Cell Viability Assays

Cell viability assays were conducted on MCF-7 and MDA-MB-231 breast cancer cell lines along with human dermal fibroblasts (HDF) as a normal cell line. Cell lines were obtained from the American Type Culture Collection (ATCC, Manassas, VA, USA). MCF-7 cells were grown in Minimum Essential Medium (MEM; Gibco, Thermofisher Scientific, Waltham, MA, USA). MDA-MB-231 cells were grown in Roswell Park Memorial Institute (RPMI) in 1640 medium (Euroclone SpA, Milan, Italy). HDF were grown in Dulbecco’s Modified Eagle’s Medium (DMEM). All media were supplemented with 10% heat-inactivated fetal bovine serum (FBS; Capricorn Scientific, Ebsdorfergrund, Germany) and penicillin-streptomycin (100 U/mL–100 µg/mL; Euroclone SpA). Doxorubicin HCl (DOX; Sigma-Aldrich, St. Louis, MO, USA) at concentrations of 0.01–100 µM was used as a positive control. The half-maximal inhibitory concentration (IC_50_) for each treatment was obtained by nonlinear regression analysis of the dose-response curves in GraphPad Prism version 7. Based on the IC_50_ values, the most active extracts were tested on HDF. For the experiment, cells were seeded in 96-well plates at 7000 cells/well and allowed to attach overnight. The next day, cells were treated with the active extracts and the cell viability assay was assessed by the MTT (3-[4,5-dimethyl-thiazol-2-yl]-2,5-diphenyltetrazoliumbromide; Sigma) assay. This assay detects the reduction of MTT by the mitochondrial dehydrogenase to blue formazan product, which reflects the normal function of mitochondria and cell viability [31]. Three independent experiments were carried out.

DMSO was used as a vehicle for the EtOH, EtOAc, and CHCl_3_ extracts, with a concentration that did not exceed 5% (in the preparation of the maximum concentration, 1000 ug/mL); to enhance the solubility of those extracts, then it was diluted with the complete culture medium to match the concentration in the extracts’ dilutions.

### 3.7. In Vivo Evaluation of the Antiproliferative Activity

#### 3.7.1. Animals

This research was carried out in accordance with accepted ethical standards. All experimental protocols at the Faculty of Pharmacy, Applied Science Private University, were approved by the Research and Ethical Committee. Female Balb/C mice, weighing 20–25 g and aged (6–8) weeks, were used acutely in the *in vivo* cytotoxicity studies. To achieve all essential environmental conditions, mice were housed in well-ventilated rooms at room temperature (25 °C) and 50–60% humidity, as well as alternate dark and light cycles every 12 h. They were housed in standard cages with a water supply.

#### 3.7.2. Acute Toxicity Test of Plants Extracts

For the LD_50_ determination of EtOH extract of *L. angustifolia*, the extract was dissolved in distilled water. Six female mice were given the EtOH extract orally in a dose of 5 g/kg. The mice were observed for 24 h for any mortality. The next dose was increased by 1.5 times if tolerated or decreased by 0.7 times in the case of mortality [32].

#### 3.7.3. *In Vivo* Tumor Inoculation and Antitumor Activity Assay

EMT-6/P (passage 3) cells were pulled out of the nitrogen tank, thawed, and cultured in 75 cm^2^ flasks containing 15 mL MEM. Cells were incubated under required conditions of 37 °C, 5% CO_2_, and 95% humidity. When cells were actively dividing and a confluent layer was formed, they were detached from the flask wall using trypsin-EDTA and PBS, incubated for 2–3 min. Afterwards, cells were washed with 5 mL MEM, transferred to a 15 mL sterile centrifuge tube, and centrifuged at 1500 RPM and 4 °C for 8 min. The formed pellet was re-suspended in 5 mL MEM, and cell counting was performed. Sixteen female Balb/C mice were subcutaneously injected with a tumorigenic dose of 150,000 cells. The animals were grouped into two groups, non-treated group (control group) and treated group, which received 500 mg/kg/day of *L. angustifolia* EtOH extract. After 10 days of tumor inoculation, tumors were measured and the treatment started. A digital caliper was used to quantify tumor volumes, commonly known as tumor sizes at the initiation of the experiment. The measurements were done in the middle of the experiment, at day 4 (day 14 after inoculation of the tumorigenic cells) and at day 7 (at the end of the experiment). A useful method that offers a quantitative evaluation of tumor growth and progression is tumor volume computation. In this case, the following equations were used to compute tumor volumes and tumor progression, respectively [33].

(2) $$V=\frac{\left(L\times W^{2}\right)}{2}$$

where V is the volume, and L and W are the length and width of the tumor, respectively.

(3) $$\%\mathrm{tumor}\;\mathrm{change}=\frac{F-I}{I}\times 100\%$$

F and I represent the final and initial tumor volumes, respectively.

### 3.8. Evaluation of Liver Functions in Control Groups

To assess the levels of the possible toxicity exerted on the liver by treatments, serum levels of liver enzymes AST and ALT were investigated for the negative control (untreated mice with tumor) and positive control (normal mice without tumor) groups. Serum samples were collected, and AST and ALT were measured using an Aspartate Aminotransferase (AST/GOT) kit and Alanine Aminotransferase (ALT/GPT) kit, respectively. The reagent was mixed according to the protocols to prepare working reagents. Working reagents were incubated at 37 °C, which is the optimal reaction temperature. In a cuvette, 50 µL of sample was mixed with 1 mL of working reagent, incubated for 1 min, and the initial absorbance was recorded. Absorbance readings were recorded also after 0, 1, 2, and 3 min. The spectrophotometer was set to read absorbance at 340 nm and was set to zero absorbance using distilled water. Working reagents were used as blanks [34].

### 3.9. Evaluation of Kidney Function

To evaluate the kidney’s level of toxicity and the risk of developing nephrotoxicity, creatinine serum levels were investigated for treated, negative control, and positive control groups. Serum samples were collected and creatinine was measured using a Creatinine Kit.

Standard (S) was provided ready for use. To make working reagents, mixtures were made in accordance with protocols and then incubated at 37 °C, which is the optimal reaction temperature. In a cuvette, 100 μL of sample was mixed with 1 mL of working reagent, and after 30 and 90 s, absorbance readings were taken. The absorbance reading on the spectrophotometer was set to 500 nm and was set to an absorbance of zero using distilled water. Working reagents were used as blanks [35].

### 3.10. Statistical Analysis

Using the SPSS statistical package version, data were averaged and presented as mean ± standard deviation (SD) of three independent experiments (version 22). To ascertain the statistical significance between the groups, one sample *t*-tests, Tukey’s, and one-way analysis of variance (ANOVA) in SPSS were utilized. When the *p*-value < 0.05, differences between groups were considered significant.

## 4. Conclusions

*L. angustifolia,* rich in phenolic substances and flavonoids, revealed promising antioxidant and antiproliferation activities. Chloroform, EtOAc, MeOH, EtOH, and aqueous extracts were tested against MCF-7 and MDA-MB-231 breast cancer cell lines. Normal fibroblast cells were used to exclude the toxic effect of the tested extracts. For the first time, the antiproliferative potential of the EtOH extract was tested *in vivo* against mice with breast cancer. Results showed a significant reduction in tumor size. Further studies are required to isolate the active constituents of *L. angustifolia* EtOH extract and study the mechanism of action of these compounds to further confirm the cytotoxic activity of *L. angustifolia.*

## Figures and Tables

**Table 1:** Total phenol contents and total flavonoids of aqueous and ethanol extracts of *L. angustifolia*.

	Total Phenol (GAE)	Total Flavonoids (mg of Rutin/g)
Aqueous extract	108.6 ± 4.96	45.25 ± 2.49
Ethanol extract	82.90 ± 10.01	50.22 ± 1.42
Methanol extract	204.87 ± 39.18	100.77 ± 2.73
Ethyl acetate extract	74.64 ± 9.66	52.50 ± 4.75
Chloroform extract	14.43 ± 2.13	3.20 ± 0.78

Data shown as average of triplicates ± SD, *n* = 3.

**Table 2:** IC_50_ values for the antioxidant activities of the plant extracts.

Plant Extract	IC_50_ (μg/mL)
Aqueous extract	138.89 ± 3.26
Ethanol extract	232.85 ± 12.32
Methanol extract	401.81 ± 12.61
Ethyl acetate extract	735.29 ± 13.10
Chloroform extract	N.A.
Ascorbic acid	5.41 ± 0.12

Data shown as average of triplicates ± SD, *n* = 3.

**Table 3:** LC-MS results of the identified compounds in the ethanol extract of *L. angustifolia*.

RT [min]	*m/z* Meas.	Identified Compound	Molecular Formula	Relative %
5.88	447.09165	Luteolin 7-O-glucoside	C_21_H_20_O_11_	29.9
6.87	359.07743	Rosmarinic acid	C_18_H_16_O_8_	37.7
28.07	227.20157	Myristic acid	C_14_H_28_O_2_	32.4

**Table 4:** IC_50_ (µg/mL) for different extracts of *L. angustifolia* tested on three cell lines (MCF7, MDA-MB-231, and Fibroblast).

	MCF-7	MDA-MB-231	Fibroblast
Aqueous	Nontoxic	Nontoxic	Nontoxic
Ethanol	104.1 ± 3.2	214.5 ± 8.3	Nontoxic
Methanol	Nontoxic	278.1 ± 3.6	Nontoxic
Ethyl acetate	1306.2 ± 12.1	94.2 ± 5.2	Nontoxic
Chloroform	30.8 ± 1.6	28.6 ± 1.1	34.05 ± 2.3
Doxorubicin	0.3083 ± 0.06	1.50 ± 0.09	0.21 ± 0.01

Results are represented as mean ± SD, *n* = 3.

**Table 5:** Effect of ethanol extract of *L. angustifolia* on tumor size (mm^3^) and weight (mg) of EMT-6/P cell line.

Group	Initial Tumor Size	Final Tumor Size	% Change in Tumor Size	Tumor Weight
**Control**	347.145 ± 97.82	551.608 ± 162.466 *	58.90%	397.75 ± 203.89 *
**Treated**	381.676 ± 34.29	216.466 ± 94.796	−43.29%	122.01 ± 110.46

Data is represented as average ± SD, (*n* = 8) * *p* < 0.01.

**Table 6:** Serum ALT, AST levels (IU/L), and serum creatinine levels in (µmol/L) for treatments, control, and normal untreated mice groups.

Group	ALT (IU/L)	AST (IU/L)	Creatinine (mg/dL)
Normal mice	62.76 ± 23.17	111.96 ± 17.28	0.10 ± 0.005
Control	90.233 ± 12.438	191.13 ± 47.62	0.11 ± 0.001
Treated	99.83 ± 4.13	266 ± 21.4	0.14 ± 0.064

Data shown as average of triplicates ± SD, *n* = 3.

## Data Availability

Not applicable.
